# A Computational Model of Cellular Mechanisms of Temporal Coding in the Medial Geniculate Body (MGB)

**DOI:** 10.1371/journal.pone.0029375

**Published:** 2011-12-16

**Authors:** Cal F. Rabang, Edward L. Bartlett

**Affiliations:** 1 Weldon School of Biomedical Engineering, Purdue University, West Lafayette, Indiana, United States of America; 2 Department of Biological Sciences, Purdue University, West Lafayette, Indiana, United States of America; Instituto de Neurociencias de Alicante UMH-CSIC, Spain

## Abstract

Acoustic stimuli are often represented in the early auditory pathway as patterns of neural activity synchronized to time-varying features. This phase-locking predominates until the level of the medial geniculate body (MGB), where previous studies have identified two main, largely segregated response types: Stimulus-synchronized responses faithfully preserve the temporal coding from its afferent inputs, and Non-synchronized responses, which are not phase locked to the inputs, represent changes in temporal modulation by a rate code. The cellular mechanisms underlying this transformation from phase-locked to rate code are not well understood. We use a computational model of a MGB thalamocortical neuron to test the hypothesis that these response classes arise from inferior colliculus (IC) excitatory afferents with divergent properties similar to those observed in brain slice studies. Large-conductance inputs exhibiting synaptic depression preserved input synchrony as short as 12.5 ms interclick intervals, while maintaining low firing rates and low-pass filtering responses. By contrast, small-conductance inputs with Mixed plasticity (depression of AMPA-receptor component and facilitation of NMDA-receptor component) desynchronized afferent inputs, generated a click-rate dependent increase in firing rate, and high-pass filtered the inputs. Synaptic inputs with facilitation often permitted band-pass synchrony along with band-pass rate tuning. These responses could be tuned by changes in membrane potential, strength of the NMDA component, and characteristics of synaptic plasticity. These results demonstrate how the same synchronized input spike trains from the inferior colliculus can be transformed into different representations of temporal modulation by divergent synaptic properties.

## Introduction

The auditory system rapidly analyzes and processes temporal modulations encoded in acoustic stimuli such as speech and animal vocalizations [1]. This temporal information is represented by patterns of neural activity synchronized to the time-varying features in the early stages of the auditory pathway. However, the precision of this temporal coding and maximal frequency of synchronization gradually decreases at successive processing stations above the auditory brainstem [1].

In the inferior colliculus (IC), which serves as the primary auditory input to the auditory thalamus, neurons fire action potentials that generally exhibit correlated rate and synchronization tuning to modulation frequency, with nearly all neurons synchronized at some modulation frequency [1]–[3]. This is not the case in auditory thalamus and cortex. Studies that recorded from the medial geniculate (MGB) and auditory cortex of marmosets [4]–[6], the MGB of cats [7], [8] and auditory cortex of rats [9] describe several main response types. Stimulus-Synchronized responses, which are similar to the IC responses mentioned above, occur for low frequency rates (<100 Hz, <50 Hz; thalamic and cortical responses, respectively), after which phase-locking rapidly diminishes with increases in frequency. Non-synchronized responses are characterized by low firing rates for low click rates, increases in firing rate during high click rates, and a notable lack of synchrony at nearly all click rates [4], [5]. Mixed responses are characterized by synchronized responses for low click rates and non-synchronized rate responses at high click rates. The response characteristics for these types are summarized in Table 1. From this data there is evidence of a transformation from temporal encoding in the IC to rate encoding in the MGB over a restricted frequency range.

**Table 1:** Characteristics of *in vivo* and *in vitro* MGB responses to periodic stimuli.

Response Type	Synchronized	Non-synchronized	Mixed
Latency	Short latency	Long latency	Short/Long latency
Synchrony	High vector strength, low pass	Low vector strength, non-synchronized	High vector strength, low-pass
Rate encoding	Low pass/band pass rate	High pass/band pass rate	Two rate regimes separated by worst modulation freq. or band pass
Source	Inherited from IC inputs	Created in MGB	Inherited from IC?
MGB Region	Generally in MGV	Generally in MGD	Generally in MGV
Putative Synaptic Source	Large IC terminals	Small IC terminals	Large and Small terminals or separate population?
Putative Plasticity	Depression	AMPA depression, NMDA facilitation (Mixed plasticity)	Facilitation or depression and facilitation

The Synchronized, Non- synchronized and Mixed categories refer to *in vivo* responses described in Bartlett and Wang [5], [6] and Lu et al. [4].

Data from brain slice studies in the rat MGB provide some evidence of the possible synaptic mechanisms of this transformation. Previous studies have revealed two populations of ascending IC inputs to the MGB [10]–[12]. The large terminal IC inputs resemble the excitatory inputs described for other sensory, “driver” inputs to thalamocortical neurons [13], [14]. These inputs have a strong, short-latency excitation that, individually, can often produce action potentials *in vitro* and exhibit synaptic depression that can last for 100-200 ms [11] thus limiting the ability of the MGB output to follow rapid temporal modulations despite phase-locked inputs [11], [15]. These inputs have mainly been observed in the ventral division of MGB (MGV) [10]–[12].

The second population of IC inputs is small-terminal excitatory inputs. These inputs have longer latencies with smaller peak amplitudes [11], [12]. Individually, these small inputs are unable to reach spiking threshold and produce action potentials *in vitro* [16]. Unlike the large terminal IC inputs, the small terminal IC inputs often exhibit facilitation and summate to generate action potentials in response to high frequency stimulation [11], [12]. Thus, these subthreshold inputs can produce action potentials in response to rapid stimulation or in the absence of inhibition. Small excitatory inputs are observed predominantly in the dorsal MGB (MGD), but can also be found in MGV. Table 1 summarizes the *in vivo* responses, their distribution in MGB and the potential *in vitro* properties associated with the *in vivo* responses.

Previous studies have shown a temporal to rate response conversion in the MGB that occurs at high frequencies, but there is a lack of understanding regarding the cellular mechanisms that produce this transformation. Table 1 summarizes the *in vivo* responses of MGB neurons to periodic stimuli, the distribution of those responses across the MGB, and the potential associated *in vitro* properties. Our model suggests possible mechanisms underlying the different responses to periodic inputs. We assessed the impact of synaptic depression and facilitation, input jitter, and membrane potential on temporal coding in a MGB thalamocortical neuron. We also determined the most sensitive parameters required in our model to either preserve temporal fidelity or transform from temporal to a rate code.

## Methods

We have focused on the ability of MGB thalamocortical neurons to transform sensory IC afferents using a detailed single compartment model. Thalamocortical neurons are relatively compact electrotonically [17], particularly within 50 µm of the soma, where most of the ascending afferents are located [10], such that use of a single compartment should be sufficient for the focus of the current study on IC inputs. The single compartment computational model is based on results from previous modeling studies [18]–[20]. This model includes a fast transient Na^+^ current (I_Na_), a delayed rectifying K^+^ current (I_K_), a persistent, depolarization-activated Na^+^ current (I_Nap_), a low-threshold Ca^2+^ current (I_T_), a high-threshold Ca^2+^ current (I_L_), a transient and depolarization-activated K^+^ current (I_A_), a slowly inactivating and depolarization-activated K^+^ current (I_K2_), a hyperpolarization-activated cation current (I_h_), a synaptic current (I_syn_), an injected current (I_inj_), and a leak current (I_leak_) (Table 2; Text S1). A Ca^2+^-activated K^+^ current (I_C_) was not included in our simulation. A previous study suggested the existence of a Ca^2+^-activated K^+^ conductance in immature MGB neurons [21] but a separate study found a Ca^2+^-activated K^+^ conductance only in the paralaminar thalamic nuclei adjacent to the MGB [22].

**Table 2:** Default Model Parameters.

Na^+^ reversal potential^1,2^	50 mV
K^+^ reversal potential^1,2^	-100 mV
Membrane leak reversal potential^1,2^	-73 mV
Max. T-type Ca^2+^ permeability (P_CaT_)^1,3^	0.00008 cm/s
Max. L-type Ca^2+^ permeability (P_CaL_)^1,2,8^	0.00001 cm/s
Max. transient Na^+^ conductance (g_Na_)^1,2^	0.01 S/cm^2^
Max. delayed rectifier K^+^ conductance (g_Kdr_)^1,2^	0.01 S/cm^2^
Max. transient, depolarization-activated K^+^ conductance (g_A_)^2,3^	0.0008 S/cm^2^
Max. slowly-inactivating, depolarization-activated K^+^ conductance (g_k2_)^2,3^	0.000134 S/cm^2^
Max. persistent, depolarization-activated Na^+^ current (g_Nap_)^2,4,9^	0.00001 S/cm^2^
Max. hyperpolarization-activated cation current (g_h_)^2,3^	0.00005 S/cm^2^
Leak conductance	0.00006.5 S/cm^2^
AMPA reversal potential^5,6,7^	0 mV
NMDA reversal potential^5,6,7^	0 mV

1 [20],^ 2^ [18], ^3^ [19], ^4^ [70], ^5^ [26], ^6^ [40], ^7^ [11], ^8^ [71], ^9^ [72]

**Figure 1:**
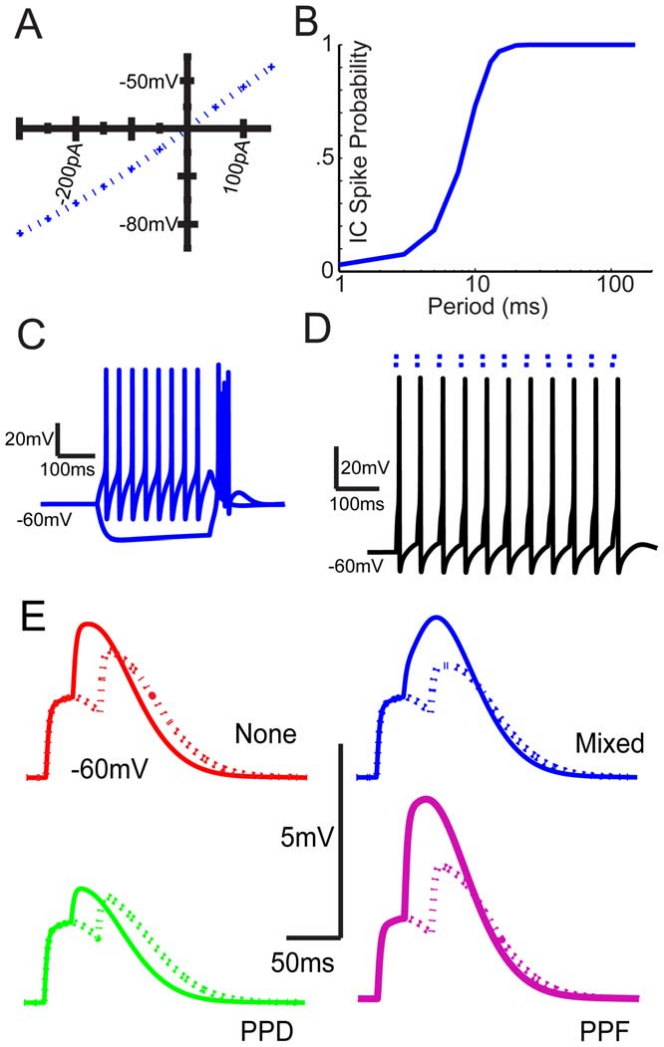
Intrinsic and Synaptic model characteristics. *A*, I-V plot of model responses to current injections. *B*, IC Input spike probability as a function of inter-click interval. Spike probabilities are close to 1 at high inter-click intervals greater than 25 ms and fall as inter-click intervals are reduced. *C,* Model responses to 300 pA and -300 pA current injection, which produces a tonic firing response and an offset burst response, respectively. *D,* Example voltage trace observed from a single trial model simulation. The dots above indicate the 2 individual spike inputs used to produce the voltage response. *E*, Voltage traces show paired synaptic inputs having interclick interval of 25 and 50 ms. Solid lines are paired EPSPs with 25 ms ICIs and dashed lines are traces with 50 ms ICIs. *From top to bottom, left to right:* Modeled paired synaptic inputs with no plasticity, AMPA and NMDA exhibiting paired pulse depression (PPD), “Mixed” inputs with AMPA exhibiting depression and NMDA exhibiting facilitation, AMPA and NMDA inputs exhibiting paired pulse facilitation (PPF).

The leak conductance was adjusted such that the resting membrane potential was approximately −68 mV, similar to measurements made in MGB neurons in brain slice preparations [16], [21]. Subsequent adjustments to membrane potential were done through constant injected current applied throughout the simulation run, at least 200 ms prior to synaptic stimulation so that the membrane potential reached a steady state. For most of our simulations we adjusted the membrane potential to −60 mV using a small bias current in order to account for the mean depolarization provided by ongoing synaptic activity, which is within the typical resting potentials recorded from MGB and VPM neurons *in vivo* [23], [24]. The model as constructed produces the typical thalamocortical tonic and burst firing responses to depolarizing and hyperpolarizing current pulses (Figure 1).

### Modeled synaptic inputs

Modeled excitatory synaptic inputs consisted of an AMPA and an NMDA component (Text S1). Synaptic conductances for each component were modeled based on previous studies [11], [25], [26] and adjusted to fit amplitude, rise and decay characteristics found in brain slice data from rat and mouse thalamic regions [11], [16], [27]–[29].

The time course of paired-pulse depression and facilitation of the AMPA and NMDA components were modeled from intracellular recordings from brain slices of rat MGB [11]. These time courses match reasonably well with another recent study of MGB neurons [12]. Depression and facilitation were incorporated into the equations for AMPA and NMDA currents through an amplitude scaling factor of the resulting excitatory AMPA and NMDA conductances. The equations and constants that govern the effects of synaptic depression and facilitation are given in the Appendix (Text S1).

The maximal conductance value for the AMPA synaptic component was varied in 10 linearly spaced steps between 0–20 nS in order to observe qualitative and quantitative differences in the response due to AMPA input conductance. This range covered the AMPA conductance measured in previous studies of thalamocortical neurons [26]. Similarly, in order to observe qualitative and quantitative differences in the response due to NMDA input conductance, the maximal conductance for NMDA was adjusted such that the ratio of NMDA to AMPA maximal conductance ranged between values of 0 to 3. For subsequent trials, specific values were chosen such that the ratio of AMPA and NMDA receptor-mediated currents were similar to those from a study of synaptic properties in ventrobasal and lateral geniculate thalamic neurons [26], [28], since the characteristics of MGB neurons were estimated initially from current clamp recordings.

The inputs to the model represent realistic *in vivo* responses from IC neurons in response to periodic click stimuli. Each IC input to an MGB neuron was modeled as a series of spike times, and a given MGB neuron could receive one or more IC inputs. Click stimuli inputs to the neuron were represented by a periodic train of inputs varied by inter-click interval. For a given IC input, the input train, or input stream, was created using a spike probability curve based on stimulus period (Fig. 1B), based on responses to sinusoidal amplitude-modulated tones in rats and click stimuli in cats [30], [31]. In addition, we adjusted each individual spike time in an input stream with an added random value taken from a Gaussian distribution with a given standard deviation in milliseconds (typically 1 ms). These values are similar to standard deviation values of IC response latencies to electrical stimulation of the cochlea in cats [32]–[34].

Each trial for a given IC input stream had a unique set of spike times determined by input periodicity, spiking probability (Figure 1) and jitter. To use numbers of trials similar to that collected during typical *in vivo* [5], [6] or *in vitro* [15] recordings, at least ten trials of a given stimulus were used to construct MGB responses simulated using a given set of parameters. Larger numbers of trials (20–50) were used when noted. Interclick intervals of click stimuli ranged from 3–150 ms, corresponding to frequencies between 6.67–333 Hz.

For subsequent simulation trials we modeled both the large-terminal and small-terminal IC inputs. Large inputs were characterized by a strong, short-latency excitation that exhibited synaptic depression. These responses can be found mainly in MGV, often without accompanying IC inhibition [11], [16]. Previous studies have shown that neurons receiving Large terminal inputs typically received 1–3 physiologically differentiated jumps in synaptic potential with increasing stimulus strength [11], [16] and exhibited synaptic depression [11], [12], [35]. We used two modeled Large inputs with a large AMPA conductance, a small NMDA to AMPA conductance ratio, and synaptic depression of both AMPA and NMDA components. In previous studies, Small inputs were characterized as exhibiting weak synaptic facilitation, longer latencies, and smaller peak amplitudes compared to Large inputs [11], [16]. We use four modeled Small inputs with a small AMPA conductance, a large NMDA to AMPA conductance ratio, and synaptic facilitation.

### Simulation

The IC input characteristics, input spike times and synaptic conductance values were written, generated and run through MATLAB (Mathworks, Inc.). Using these parameters, MGB neuron model simulations were performed in the NEURON simulation environment [36]. Analysis was done in MATLAB. All computation and analysis were performed on DELL workstations using the MS Windows XP operating system. The simulation trials used an integration time step value dt = 0.02 ms. This value was empirically verified to be sufficient to simulate accurate ion channel and synaptic currents.

### Data analysis

We focused mainly on suprathreshold responses since one goal of this study was to reveal the biophysical mechanisms underlying MGB *in vivo* responses. Unless noted, spike count and firing rate was computed from the entire stimulus duration. The ability to synchronize to a click train was quantified by measuring the vector strength (VS = (1/n)*√((Σcosϕ_i_)^2^+(Σsinϕ_i_)^2^), where n = total number of observed spikes, ϕ_i_ = phase of observed spike relative to inter-click interval) of the model response at each inter-click interval. Statistical significance was assessed using the Rayleigh statistic 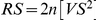, which considers the number of evoked spikes [4], [5]. A threshold Rayleigh statistic value of 13.8 was considered statistically significant (P<0.001) [37]. The time window for Rayleigh computation was 50 ms following stimulus onset through the duration of the 500 ms stimulus since we focus on the sustained and not the onset responses to our input stimuli, similar to previous studies [5], [38].

## Results

### Model

The MGB neuron model faithfully reproduced the tonic and burst modes of firing observed in thalamocortical neurons in response to depolarizing and hyperpolarizing current pulses (Figure 1A, 1C). Nearly all simulations were run with the membrane potential set to −60 mV. The calculated input resistance of the neuron model from a membrane potential of −60 mV was approximately 75 MΩ, which is within the range of MGB neurons recorded *in vitro* [16] and thalamic neurons *in vivo* [23].

The model responses to single synaptic events were comparable to intracellular MGB responses to electrical synaptic stimulation. The AMPA and NMDA components of the EPSP were comparable to those recorded intracellularly [12], [16], [39]. Four different variations of short-term plasticity were compared in this study. Equations describing this plasticity are given in the Appendix (Text S1). First, for the None condition, AMPA and NMDA conductances were used without short-term plasticity, meaning no paired pulse depression or facilitation (Fig. 1E, red traces). Therefore, the conductances had no dependence on input spiking history. Second, for the Depression condition, both AMPA and NMDA components were modified to exhibit interspike-interval dependent synaptic depression (Fig. 1E, green traces). Third, for the Facilitation condition, AMPA and NMDA components both exhibited interspike-interval dependent synaptic facilitation (Fig. 1E, purple traces). The last variation used an AMPA component exhibiting paired pulse depression coupled with an NMDA component exhibiting paired pulse facilitation, because previous results suggested that this may occur in some MGB neurons (Fig. 1E, blue traces) [11]. These four types were named “None”, “PPD”, “PPF”, and “Mixed”. The resulting EPSPs are shown in Figure 1E for an interclick interval of 25 or 50 ms.

### Influence of synaptic parameters

**Figure 2:**
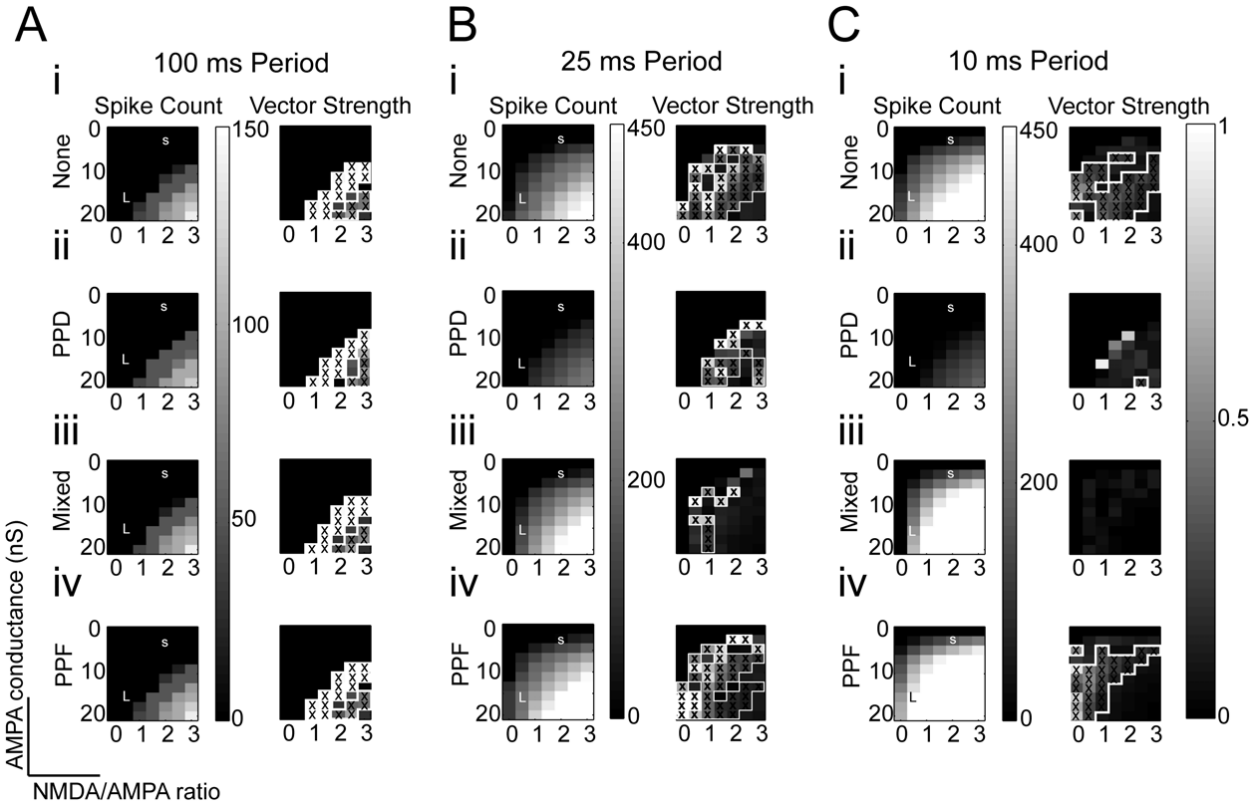
Model response characteristics produced as a result of adjusting AMPA and NMDA conductance levels. Y-axis values correspond to AMPA conductance (0 – 20 nS), X-axis values correspond to ratio of NMDA to AMPA conductance (0, .5, 1, 1.5, 2, 2.5, 3). Values of AMPA conductance and NMDA:AMPA ratio chosen for Large and Small IC inputs are identified by a white L and S on the 100 ms color plots, respectively. Regions outlined in white indicate regions where response is statistically calculated to be synchronized (Rayleigh statistic >13.8, p<0.001). As ISIs decrease, the regions of synchronized responses diminish. *A, i-iv:* Spike rates and vector strength plots for 10 simulation trials at 100 ms interclick intervals, 500 ms duration each trial. *From top to bottom:* Spike rate and vector strength plots for no plasticity, short-term depression, mixed (AMPA depression + NMDA facilitation), and short-term facilitation do not vary much at large ISIs. *B, i-iv*: Spike rates and vector strength plots for 10 simulation trials at 25 ms interclick intervals. Synaptic facilitation increases spike rate, while synaptic depression decreases overall spike rates. *C, i-iv*: Spike rates and vector strength plots for 10 simulation trials at 10 ms interclick intervals. Short-term plasticity greatly affects overall spike rate. With the exception of one conductance pair in the PPD case, regions of synchronized responses only occur in simulations with no plasticity or synaptic facilitation.

In order to assess the basic dependence of firing rate and synchrony on excitatory conductance values (i.e. the magnitude of the EPSP), the AMPA and NMDA maximum conductances and the NMDA/AMPA peak conductance ratio were varied such that the AMPA maximum conductance was set to linearly spaced values between 0 and 20 nS and the NMDA/AMPA peak conductance ratio corresponded to the following values: [0, .5, 1, 1.5, 2, 2.5, 3]. Simulation trials were run using a single modeled input at each pair (70 pairs total) of AMPA conductance and NMDA/AMPA ratio values. The spike rate and vector strength for each parameter pair are shown in Figure 2. Regions of synchronized responses are indicated by white borders and ‘x’s on the corresponding vector strength plots. At 100 ms interclick interval (ICI), the addition of short-term plasticity had little effect on spike rate and regions of synchronized responses were similar. At each conductance value pair for 25 ms ICI, higher spike counts were observed in the Facilitation condition, followed by Mixed and None, while PPD generated much smaller spike counts. At 25 ms ICI, firing rates for all types of plasticity increased in an orderly matter as g_AMPA_ and the NMDA/AMPA ratio increased, and this increase was more pronounced for Mixed & PPF. For both 100 and 25 ms ICI, a band of high synchrony (vector strength near 1) was apparent in the vector strength plot, running diagonally from high g_AMPA_ and low NMDA/AMPA ratio to a low g_AMPA_ and high NMDA:AMPA ratio. These parameters only enabled a brief window of suprathreshold excitation on each cycle to maintain the high vector strength. When both g_AMPA_ and the NMDA/AMPA ratio were high, vector strength declined. For 25 ms ICI, a high NMDA/AMPA ratio desynchronized MGB responses at all conditions except PPD, where it assisted in maintaining response synchrony by maintaining a sustained depolarization to keep the neuron near threshold. At 10 ms ICI, which produced maximal firing rates in the model, a larger range of spiking was observed due to summation of inputs. Facilitation and Mixed conditions produced spiking at nearly every conductance value pair and produced spike rates up to 90 spikes/sec (>40 spikes/trial), several times larger than observed at 50 and 100 ms ICI. Depression greatly reduced spike rates to ≤20 spikes/sec. At 10 ms, low spike counts hampered response synchrony when there was synaptic depression, and synchrony was not observed in the Mixed condition. Synchrony was observed for the None or Facilitation conditions, but it was strongest from low NMDA/AMPA ratios (Figure 2C).

For subsequent simulations we chose sets of values that characterized the putative “Large” and “Small” terminal IC inputs in terms of response magnitude, NMDA receptor contribution, and short-term plasticity. The Large and Small IC inputs are also intended to correspond to the main IC excitatory inputs to MGV and MGD, respectively (Table 1). These are indicated by the letters “L” and “s” in the spike count plots of Figure 2. For the large terminal IC inputs, we chose conductance values of individual inputs that corresponded to a large AMPA peak conductance value (16 nS) and a moderate NMDA/AMPA current ratio (.5), similar to what has been reported in retinogeniculate synapses [26], [28], [40]. Note that near resting membrane potentials, the effective NMDA/AMPA ratio will be smaller. For small terminal IC inputs, we chose conductance values that had a relatively smaller AMPA peak conductance (2 nS) and a high (2) NMDA/AMPA current ratio (Marked by “L” and “s” for Large and Small Inputs, respectively, on Figure 2). The conductance plots in Figure 2 were taken from simulations with a single afferent input. A study of the cortical and collicular terminals in the rat MGB [10] suggested that differing proportions of the large and small IC synaptic terminals were found between MGV and MGD. A previous study [16] indicated that MGB neurons receive convergent input and there is evidence suggesting that there are numerous, non-lemniscal small terminals in MGB [10], [12]. Therefore, we segregated inputs corresponding to large-terminal IC inputs to MGV from those corresponding to small terminal IC inputs to MGD. The large terminal inputs are relatively sparse, all or none inputs, so we chose to use only 2 inputs for Large inputs. By contrast, Small inputs were modeled as more convergent, with 4 inputs to a given neuron, in line with previous measurements [10]. The degree of depression or facilitation for EPSPs in a given input train was dependent only on the timing of the EPSPs in their specific input train and was not affected by stimuli from other input trains. For the 1 ms temporal jitter used for individual inputs, increasing the number of IC inputs produced similar effects to increased synaptic conductance and elevated spike rates (data not shown).

### Influence of synaptic input jitter on model output

**Figure 3:**
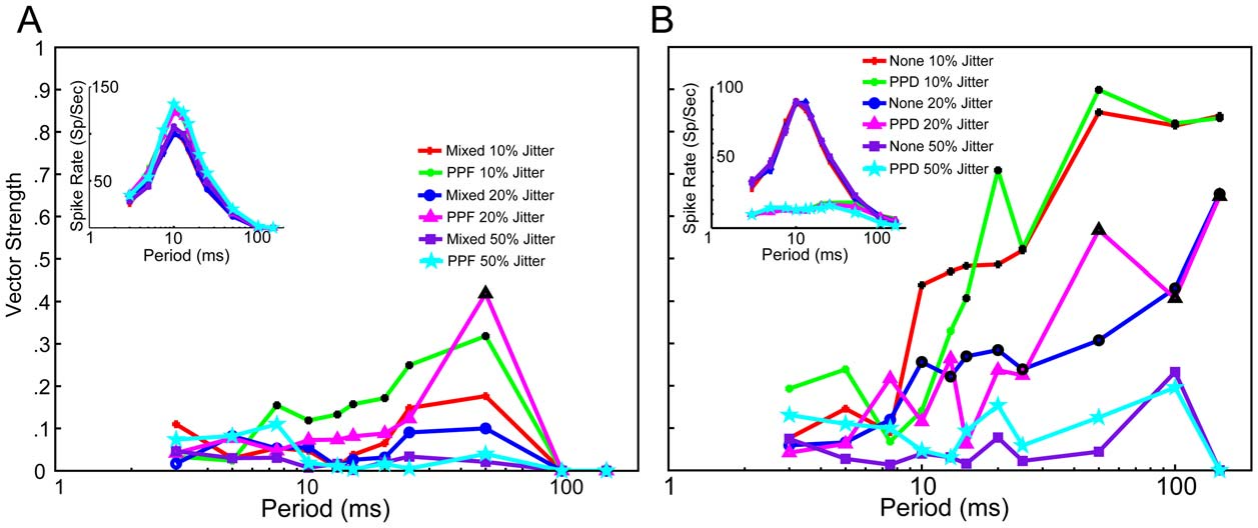
Large jitter values relative to period length desynchronize model responses. *A,* Vector strength plot for 4 Small inputs exhibiting AMPA synaptic depression and NMDA synaptic facilitation (Mixed) or AMPA and NMDA facilitation (PPF). Red dots, blue circles and purple squares indicate Mixed input responses with jitter scaled to 10%, 20% and 50% of ICI, respectively. Green dots, pink triangles, and blue stars indicate PPF input responses with jitter scaled to 10%, 20% and 50% of ICI, respectively. Synchronized responses are found in the PPF case with 10% and 20% jitter at ICIs indicated with black symbols. *Inset:* Spike rates for the parameters described in *A.* Changes in jitter had little effect on spike rates in the PPF and Mixed case. *B,* Vector strength plot for 2 Large inputs, exhibiting either synaptic depression (PPD) and no plasticity (None). Red dots, blue circles and purple squares indicate “None” input responses with jitter scaled to 10%, 20% and 50% of ICI, respectively. Green dots, pink triangles, and blue stars indicate PPD input responses with jitter scaled to 10%, 20% and 50% of ICI, respectively. Vector strength increases with reduced jitter, as do the regions of synchronized activity (indicated with black symbols) *Inset:* Rate curves of the parameters described in *B.* Changes to input jitter do not affect spike rate curves.

**Figure 4:**
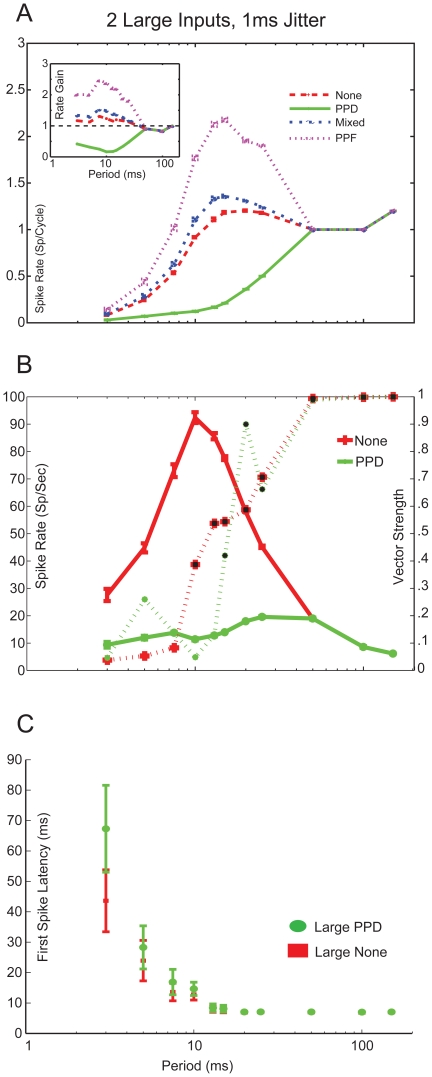
Large inputs exhibiting PPD produce synchronized, low-pass responses. *A,* Spike rate curves from simulations using 2 Large inputs with 1 ms jitter. Rates are given in spikes/cycle. *Inset*: Rate gain curves calculated from model spike rates and input spike times. Spike rates do not change with plasticity at ICIs≥50 ms. PPF inputs (purple) greatly increases rate gain, Mixed inputs (blue) slightly increases gain. Inputs exhibiting PPD (green) reduces gain. *B,* Spike rate (solid lines) and vector strength (dashed line) curves of 2 Large inputs. Red lines with “+” have no added synaptic plasticity, green lines indicate responses using PPD. Synchrony is indicated with black symbols. *C,* Measured First Spike Latency (FSL) of the Large PPD (Green ovals) and Large None (Red “+”) responses. At ICIs>10 ms, FSL is measured to be ∼7 ms, after which FSL increases due to decreased spike probability.

**Figure 5:**
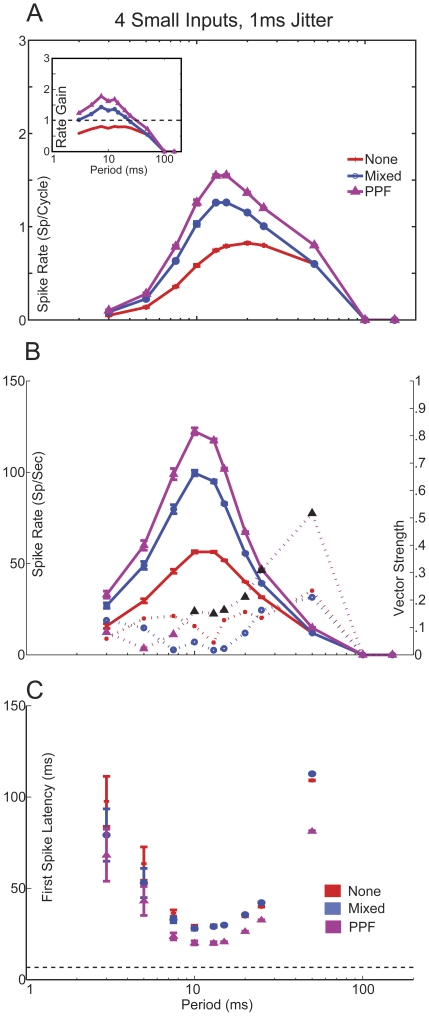
Small inputs exhibiting either PPF of Mixed plasticity produce band-pass, non-synchronized responses. *A,* Spike rate curves from simulations using 4 Small inputs with 1 ms jitter. Rates are given in spikes/cycle. Simulations with PPD produced subthreshold activity. *Inset:* Rate gain curves calculated from model spike rates and input spike times. Spike rates do not change with plasticity at ICIs≥100 ms. Purple lines with triangles mark PPF, blue lines with circles indicate Mixed, and Red lines with plus symbols indicated no synaptic plasticity. *B,* Spike rate (solid lines) and vector strength (dashed lines) curves of 4 Small inputs. Synchronized responses were only found in the PPF case between 10 and 50 ms ICI, marked in black symbols. *C*, FSL curves for Small None, Mixed and PPF inputs. Dashed line shows FSL curve of 2 Large PPD inputs for comparison. Subthreshold responses were observed at ICIs≥100 ms and therefore no FSL was measured. FSL values reduced with ICI until a minimum is reached at an ICI of 10 ms, after which FSL increases due to decreased input spike probability.

A major factor that will influence the preservation of synchrony from the IC to the MGB neuron is the timing variability of inputs. Electrical stimulation of the IC has suggested that IC timing variability is low for stimulation in the central nucleus [32]–[34], but may be higher for the non-primary, longer-latency IC pathways that will project to MGD. In addition, there is convergence of inputs from IC to MGB, such that neurons responding with different latencies may converge on the same neuron, which would lead to additional timing jitter [34], [41]. We adjusted the degree of synaptic input timing variability to determine its effects on response synchrony measured using vector strength and the Rayleigh statistic. For Figure 3, jitter values were normalized as a percentage of interclick intervals. Although this gave large differences in absolute timing at long ICI, expressing jitter as a proportion of the interclick interval provided a better understanding of the sensitivity to timing variation. Changes in rate and synchrony for a fixed jitter value of 1 ms can be seen in Figs. 4 and 5. We constrained our trials to runs with 4 Small inputs exhibiting either Mixed or PPF plasticity (Fig. 3A) or 2 Large inputs exhibiting no plasticity or PPD (Fig. 3B). 4 Small inputs exhibiting AMPA depression and NMDA facilitation (Mixed case) produced non-synchronized responses regardless of jitter. Similar runs using the PPF case produced synchronized responses at an ICI of 50 ms for 20% jitter and at ICIs between 7.5 and 50 ms for 10% jitter. This region of synchrony was similar to simulations using 1 ms jitter, which produced synchronized responses between 10 and 50 ms. Input jitter had almost no effect on average firing rates for either Large or Small inputs, although they were sensitive to changes in plasticity (Figure 3A)

10% jitter produced synchronized regions for ICIs≥15 ms in the PPD case and for ICIs≥10 ms in the None case. Increasing the jitter to 20% produced synchronized regions for ICIs≥50 ms in the PPD case and for ICIs≥10 ms in the None case, albeit with reduced vector strength. As expected, high jitter to period ratios (50%) typically produced a non-synchronized response. The regions of synchronized responses for 10% jitter were similar to those observed in simulations using 1 ms jitter for each period (Figure 4B). Interestingly, even very low input jitter (<1 ms for ICI<10 ms) was unable to produce significant MGB response synchrony, despite MGB firing rates >30 spikes/sec.

### Large inputs

Several physiologically plausible response properties emerged when the synaptic properties of the Large inputs were considered. Figure 4A displays the difference between IC input spike rates and MGB output spike rates, in term of spikes/cycle or rate gain (inset), using Large inputs for each plasticity type. There were no differences in rate gain at ICIs ≥ 50 ms, because short-term plasticity was relatively weak at these intervals for sensory inputs. As the interval decreases, plasticity had a stronger effect on the model output rate.

For the simulations using synaptic depression, the response rate and vector strength curves showed synchronized responses at ICIs≥15 ms and increased in rate with lower ICIs up to 25 ms, after which rates decreased with lower ICIs. This response is similar to the stimulus-synchronized responses observed in several *in vivo* studies, where there is a region of synchronized responses at which rate increases with stimulus frequency until a limit where synchrony fails and spike rates are drastically reduced. In comparison, simulations that used no synaptic plasticity produced larger overall rates that peak at 10 ms and produced synchronized responses at ICIs≥10 ms (Figure 4B).

Based on non-synchronized responses observed *in vivo* [5] and *in vitro* [11], we expected that the observed first spike latency (FSL) relative to stimulus onset for Large inputs would be small (Table 1). We tested this hypothesis by measuring the timing of the first evoked spike relative to the stimulus onset for the simulated data collected using the parameters described above. For ICIs≥20 ms, FSL remained constant at or near a value of 7 ms. At lower ICIs, FSL grew and varied with decreasing intervals due to decreases in input probability and synaptic depression (Figure 4C). The absolute latencies are somewhat longer than expected for the Large inputs. This is potentially due to the −60 mV resting potential used or the presence of a transient A-type potassium current that resists transient depolarizations [42]. Under conditions of high arousal and sustained depolarized membrane potentials *in vivo* or coactivation of many input fibers simultaneously by electrical stimulation, one would expect a shorter latency.

### Small inputs

Small inputs are characterized by exhibiting weak synaptic facilitation and having longer latencies and shorter peak amplitudes compared to Large inputs. This is believed to be due to a reduction in AMPA receptor-mediated currents and an increase in NMDA receptor-mediated current. These inputs can be found in both MGV and MGD but are found in higher proportions in the dorsal division. Corticothalamic inputs would have similar properties but with much stronger facilitation and over a much broader time window. Typically, neurons receiving the small inputs also receive IC inhibition, but we have chosen to isolate the transformations provided by the excitatory inputs here. We discuss the possible role of inhibition in shaping temporal response in the Discussion. Although previous studies have shown short-term synaptic facilitation for these inputs [11], [12], it is currently unclear whether it is the NMDA component alone or both AMPA and NMDA components that exhibit facilitation. Therefore we ran separate simulations in which we used either the None, PPF or Mixed plasticity for the Small inputs.

Figure 5A displays the changes in spikes per cycle and the changes in rate gain between calculated IC input and MGB output spike rates using Small inputs for each of the four plasticity types. At ICIs ≥ 100 ms, the EPSPs were unable to reach spike threshold. Mixed and PPF inputs increase the rate at lower ICIs compared to inputs without plasticity.

Regardless of plasticity, each simulation produced similar shaped rate responses, with the PPF and Mixed having larger overall rates except for ICIs≥100 ms, at which all responses were subthreshold. PPF responses were synchronized for a range of ICIs between 10 – 50 ms, while the Mixed response produced non-synchronized responses at all ICIs tested. Simulations without synaptic plasticity produced the lowest overall rate curves and were generally non-synchronized except at an ICI of 20 ms (Figure 5B).

For each of plasticity type tested, the measured FSL at each ICI showed similar trends. At ICIs≥100 ms, where only subthreshold responses were observed, no FSL could be measured. At all other ICIs, FSL decreased to a minimum value of 28.8 ms, 20.2 ms, and 28 ms for Mixed, PPF and None cases at 10 ms ICI, respectively. This was much higher than the latencies observed for the Large inputs, because the Small inputs required multiple cycles of EPSPs to reach threshold. For ICIs≤10 ms FSL values rose, which was likely due to decrease in IC input probability causing fewer inputs to summate and reach spike threshold (Figure 5C).

### Role of NMDA-dependent synaptic component on model response

**Figure 6:**
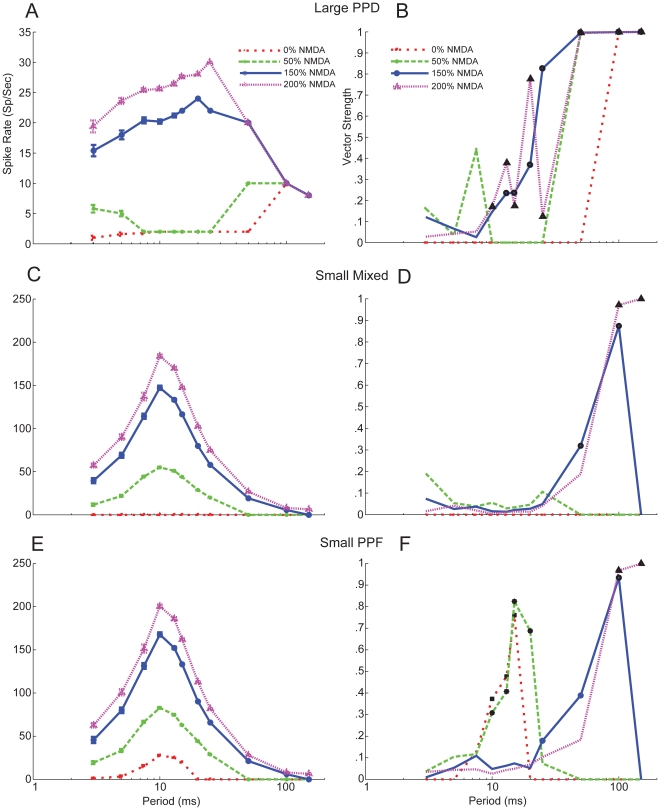
Reduction of NMDA receptor mediated current shifts responses rates and synchronization regions. *A,* Model rate responses for 2 Large PPD Inputs with 1 ms jitter. Red lines show 0% NMDA, green lines show 50%, blue lines show 150% and purple lines show 200% NMDA. *B,* Vector strength curves for the same parameters as in *A.* Reduced NMDA reduces regions of spiking, which occurs at high ICIs where the effects of synaptic depression diminishes or at low ICIs where summation of EPSPs may occur. At low ICIs, typically only an onset response is observed followed by weak sustained or subthreshold activity. Synchronized responses are indicated with black symbols. *C,* Model rate responses for 4 Small Mixed Inputs with 1 ms jitter. Colors and symbols indicate percent of NMDA conductance used as described in *A*. *D,* Vector strength curves for the same parameters as in *C*. Synchronized responses are found only at high ICIs with increased NMDA. *E*, Model rate responses for 4 Small PPF Inputs with 1 ms jitter. *F,* Vector strength curves for the same parameters as in *E.* Although high vector strength values are observed at ICIs≥100 ms for trials with increased NMDA, these responses produce small spike rates and were not found to be synchronized. Reduced NMDA does produce synchronized responses between ICIs of 10 – 20 ms.

Given the strong dependence of rate on ICI and synchrony on jitter as proportion of ICI, one major factor that can potentially influence both of those measures is the magnitude of the NMDA-dependent component of the EPSP [30], [43]. In Figure 6, rate and vector strength responses were measured during simulations where we varied NMDA conductance.

At interclick intervals≥100 ms, spike rates and vector strength for the Large PPD responses were not affected (Fig. 6A,B). Halving the NMDA component reduced the spike rate, producing an onset response for ICIs between 7.5 and 25 ms. Simulations without NMDA produced only onset responses for ICIs≤50 ms (Figure 6A). Increasing the NMDA conductance elevated spike rates at ICIs≤100 ms and extended the synchronization boundary to 12.5 ICI for 150% and to 10 ms for 200% NMDA (Figure 6B). From these data, it appears that the NMDA component of Large IC excitation contributes strongly to the maintenance of sustained MGB firing and synchronized responses for short ICIs, rather than a desynchronizing depolarization.

For Small inputs, whether the plasticity was Mixed or PPF, a significant NMDA component was required for the inputs to reach threshold (Fig. 6C,E). For Mixed responses, increasing NMDA allowed the long ICI responses to become suprathreshold and produced synchronized responses. For PPF responses, synchrony was bandpass in the 7.5 – 20 ms range when NMDA was reduced or absent because there was a sustained rate response. Increasing NMDA for PPF responses resulted in synchronized responses only at long ICI. Halving or removing NMDA conductance in both the Small Mixed and PPF cases reduced the range of spike responses. At 50% NMDA, spike responses were observed only for ICI<50 ms. Increasing the NMDA conductance enhanced rate responses of Small input similarly for Mixed and PPF responses (Figure 6C, E). The magnitude of the NMDA component controlled the high-pass filtering characteristics of the Small inputs, such that the maximum ICI at which spiking occurred was 150 ms, 100 ms, and 50 ms for 150%, 100%, and 50% NMDA, respectively (Fig. 6C,E). These results suggest that for Small inputs, a relatively large NMDA/AMPA ratio (∼2) is needed to obtain responses approximating those observed physiologically in areas targeted by Small inputs, such as the MGD [11], [16].

### Influence of membrane potential

Nearly all of our simulations were run at a resting membrane potential of −60 mV. However, membrane potential can vary between neurons and can be modulated through various means, including metabotropic glutamate receptors [11], [44] and neuromodulators [45], [46], which can affect the resulting neural response. We examined the effect of membrane potential on the rate and synchrony responses of our model by running the model with either 2 Large or 4 Small inputs with 1 ms jitter at different membrane potentials. A bias current was added and adjusted such that the resting potential ranged between −55, −65, and −75 mV at 200 ms after onset of the bias current, when the neuron and its intrinsic conductances had reached a steady-state.

**Figure 7:**
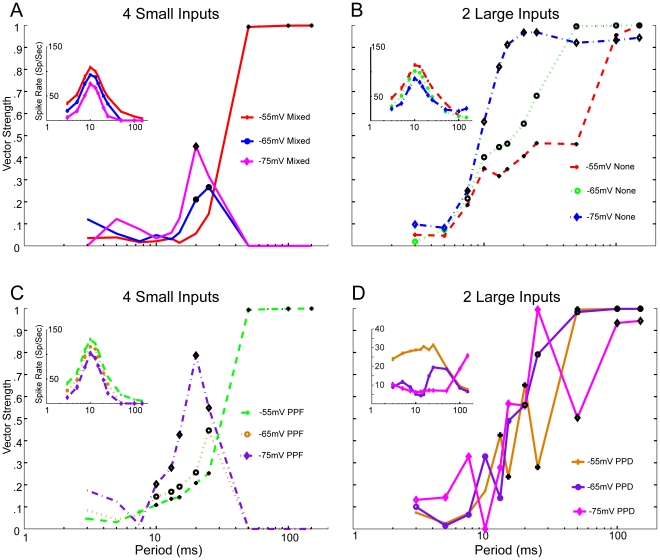
Changes in membrane potential affect response rates and synchronization boundaries. Plus, circle and diamond symbols indicate simulations run at -55 mV, -65 mV, and -75 mV, respectively. *A,* Vector strength responses for trials using 4 Small Mixed inputs with 1 ms jitter. At -55 mV, synchronized responses were observed at ICIs≥50 ms. At more hyperpolarized membrane potentials, responses at these ICIs were subthreshold. Synchronized responses are observed at 20 – 25 ms. *Inset:* Spike rate responses for parameters used in *A*. At more depolarized membrane potentials, response rate is elevated. At hyperpolarized membrane potentials, rate is reduced. *B,* Vector strength responses for trials using 2 Large inputs and 1 ms jitter without synaptic plasticity. Synchronized responses were found at ICIs≥8.5 ms at all membrane potentials. *Inset:* Spike rate responses for *B. C,* Vector strength responses for 4 Small PPF Inputs with 1 ms jitter. Responses are synchronized at ICIs between 10 – 25 ms at all membrane potentials used. At -55 mV, synchronized responses are found at ICIs≥50 ms. At these ICIs responses are subthreshold at -65 and -75 mV.

In Figure 7A–C, which included Small Mixed, Small PPF, and Large responses without plasticity, MGB responses were dominated by sustained responses for ICIs<50 ms. The rates vs. ICI in these cases were similar in shape, with higher rates at more depolarized membrane potentials. For Small inputs, membrane potential dictated the minimum ICI at which spiking responses were present, with no responses for ICIs ≥ 50 ms at V_mem_ = −65 or −75 mV.

Large PPD inputs were strongly affected by V_mem_. At −75 mV, when the neurons were in the burst firing mode, synchronized responses were observed for ICI ≥ 100 ms. Onset responses were only observed for ICI≤50 ms. At −65 mV, sustained responses were present for ICI≥20 ms and were synchronized. At −55 mV, sustained responses were present at all ICI and were synchronized for ICIs≥12.5 ms.

## Discussion

### Summary of results

This study modeled realistic synaptic IC input trains exhibiting different types of short-term plasticity and used these inputs to drive a biophysical model of a thalamocortical MGB neuron. Several parameters that contribute to maintaining synchronized responses from IC or transforming synchronized IC inputs to non-synchronized and Mixed MGB responses were investigated. The effects of short-term plasticity on firing were evident for ICIs<50 ms (>20 Hz). PPD reduced the gain and Mixed or PPF plasticity increased the MGB rate gain relative to IC inputs (Figs. 4A, 5A). Response synchrony was maintained by generating suprathreshold excitation with an appropriately sized NMDA component (Figs. 2,6). IC inputs were segregated into Large PPD for MGV inputs and Small Mixed or Small PPF inputs for MGD inputs. Large PPD inputs produced synchronized, low-pass synchrony responses and low overall firing rates with short-latency responses (Fig. 4). By contrast, Small inputs produced long-latency, band-passed rate responses with no response to long ICI, but high peak rates at 10 ms ICI. Despite high response rates, responses to Small Mixed inputs were non-synchronized at all ICI, whereas Small PPF responses were often synchronized (Fig. 5). The modeled MGB responses demonstrate how short-term plasticity produces unique transformations of IC input with different emphasis on long versus short ICI representations in the corresponding MGB target cells.

### Model considerations

We chose a single compartment model for computational efficiency and to focus on the excitatory IC afferents, which show different sets of properties and distributions within the rodent MGB [11], [12]. Thalamocortical neurons are electrotonically compact [17], [47], [48], particularly within 50 µm of the soma, where many of the ascending afferents are located [10]. Feedforward IC inhibition, feedback TRN inhibition, and interneuron inhibition were not included in order to examine the transformations produced by excitatory afferents. Many neurons receiving Large IC inputs, mainly in MGV, do not receive feedforward GABA_A_ inhibition so these would not be affected [11], [12], [16]. Nearly all MGV and MGD neurons receiving Small IC inputs also received IC inhibition. Inhibition, neuromodulators or intrinsic currents that hyperpolarize an MGB neuron below −60 mV could potentially suppress responses to high frequency inputs. Some MGB neurons receive feedforward GABA_B_ inhibition [16], which could inhibit firing, as shown in a computational model [49]. The effects of inhibition are similar to those that could result from membrane potential hyperpolarization (Fig. 7A,C).

Our model simulations were typically run with an initial membrane potential of −60 mV, which is well within measured values taken from intracellular brain slice recordings [12], [16], [21], [39] and *in vivo* recordings [24]. Castro-Alamancos [15] found that significant synaptic depression at input frequencies >10 Hz often rendered the thalamic responses subthreshold. In the presence of acetylcholine and norepinephrine, the neurons depolarized and could follow inputs with frequencies up to 40 Hz, similar to the current study. Therefore, lasting changes in membrane potential by activation of metabotrophic glutamate receptors [11], [44], GABA_B_ receptors [11] or neuromodulators [45], [46] will be important regulators of synchronization boundaries of Large and Small inputs as well as the rate boundaries of neurons receiving Small inputs.

Although the focus was on the sensory IC inputs, layer 6 corticothalamic feedback would be expected to appear as a more extreme version of the Small PPF inputs from IC. Corticothalamic inputs throughout the thalamus exhibit potent facilitation over a wide range of interspike intervals, with much stronger and longer lasting facilitation than from IC [11]. Unlike the high synchrony and firing rates found in IC afferents, layer 6 corticothalamic afferents often have lower rates that are not obviously stimulus-locked [50]. Large layer 5 corticothalamic feedback has been shown to have synaptic properties similar to the Large PPD IC inputs in MGV [35].

### Effects of synaptic input jitter

We modeled input jitter using inputs whose latencies were individually fairly precise (1 ms jitter), based on studies of electrical stimulation of the IC. Lumani and Zhang [41] found that responses to tone stimuli in the dorsal cortex of the IC (ICd) had longer and much more variable first spike latencies compared to those in central nucleus of the IC (ICc). This could lead to longer, variable first spike latencies in MGD neurons [51], [52], as well as ongoing phase differences between IC inputs. MGD neurons are also where the higher proportion of non-synchronized responses have been observed [6]–[8]. Although large jitter relative to ICI can produce non-synchronized responses (Figure 3), this was not a necessary condition. Small PPF and especially Small Mixed inputs with 1 ms jitter could sufficiently produce non-synchronized responses in MGB outputs (Fig. 5).

### Temporal filtering via differential short-term plasticity

Synaptic depression and facilitation have been posited to act as temporal filters. Synaptic depression serves as a low pass filter of inputs, allowing transmission of low frequency inputs while attenuating and suppressing spike rate responses at high frequencies [53]. Depression suppresses the influence of sustained inputs that are often representing the ongoing presence of a set of stimulus features. Therefore, synaptic depression may be a cellular correlate of stimulus-specific adaptation in the thalamus, which would be input specific [54], [55]. Synaptic facilitation has an opposite effect. Typically, small EPSPs that exhibit facilitation are subthreshold initially even when multiple inputs are coactivated. Therefore, summation of high frequency inputs produces a high pass filter and can promote the generation of an ICI-dependent rate and latency response. Additionally, sustained inputs are preserved as a rate code, while onset activity is suppressed [56]. Short-term plasticity may contribute to the segregation of onset and sustained activity and to the integration of multiple smaller inputs over a longer time-scale.

### Effects of short-term plasticity – comparison with previous studies

Lateral geniculate (LGN) neurons receive retinal inputs whose AMPA and NMDA components exhibit depression [40], [43]. Bartlett and Smith [11] also showed that isolated AMPA and NMDA EPSPs depressed for large inputs. The current study assumed that the magnitude of depression in both the AMPA and NMDA components was equal, except in the Mixed case. The current study did not attribute depression to any specific mechanism, but retinogeniculate synapses exhibit desensitization [40] and share morphological and physiological characteristics with the Large IC inputs.

Although a strong NMDA component can desynchronize responses (Fig. 2), especially at high frequencies, the sustained depolarization it produces increased a neuron's sensitivity during sustained activity similar to what has been observed in rat LGN neurons [43]. By elevating the amplitudes of inputs to become suprathreshold, the NMDA component can also maintain the transmission of weaker synchronized inputs by boosting their response (Fig. 2,6). The different NMDA/AMPA ratios ascribed to Large and Small inputs may be comparable to non-lagged and lagged cells observed in the LGN, respectively [57].

### Transformation of information from IC to MGB

We chose to model the responses to click stimuli in order to follow more closely the results of Bartlett and Wang [5] and electrical stimulation in brain slice studies [11], both of which consist of discrete, periodic stimuli and which produce synchronized and non-synchronized responses. Certainly, a key transformation that occurs in the IC is the generation of strong band-passed rate tuning in many IC neurons in response to SAM stimuli, resulting mainly from a reduction in the number of spikes per cycle at lower modulation frequencies [58]. Although the rate coding observed in the MGB and our model could be inherited via inputs from IC, responses to periodic and AM stimuli in IC consistently produce phase locked responses up to a stimulus AM frequency of 300 Hz [58]. While IC rate responses may be recreated at the IC – MGB synapse with weak or absent MGB synaptic plasticity, our model has shown that synaptic depression and facilitation reduced or enhanced rate gain, respectively, relative to IC inputs for spike rates >20 Hz. In addition, we demonstrated that, unlike the IC, there is a complete dissociation between strong firing rate and response synchrony, even at low modulation frequencies, for MGB neurons with Mixed plasticity.

### Functional implications

In brain slice studies and *in vivo* [5], [11], [59], Large inputs and their accompanying synchronized responses are predominantly found in the MGV, although approximately one-quarter of MGV neurons responded with non-synchronized responses [6]. In contrast, non-synchronized responses are prevalent in the MGD [6]. A recent study [12] determined that the MGD received mixed excitatory and inhibitory inputs from the lateral cortex of the IC (ICl) that also exhibited synaptic facilitation. Conversely, neurons in the MGV received purely excitatory inputs from ICc that exhibited synaptic depression. The authors concluded that the ascending pathway from ICc to MGV exhibited “driver” properties, while the pathway from ICl to MGD exhibited “modulator” properties [12]. Our results examine the consequences of these two input types for temporal processing. The synchronized outputs from MGV preserve the phase-locked inputs from IC, thus preserving temporal information en route to cortex. The MGD is considered one of the “higher-order”, intergrative sensory thalamic nuclei [13], presumably acting to sculpt complex auditory responses or as an information pathway linking differing layers of cortex [60]–[62]. Our model suggests that the non-synchronized outputs from MGD appear to be a rate code transformed from phase-locked inputs from ICl neurons [63].

The transformation from a temporal to rate code in the MGB would most likely impact neural processing of rapid time-varying features of acoustic signals in the frequency range of 50–500Hz, which are typically synchronized in IC but not as often in MGB [1], [5]. In humans, this range has been implicated in processing of features in speech, including pitch, voicing, stress and intonation [64]. This frequency range also corresponds to components of species-specific vocalizations in animals such as marmosets [65], guinea-pigs [66], and rats [67]. The MGB models of synaptic depression and facilitation are easily adaptable and can be used to investigate the role of plasticity in shaping responses in other neural regions, such as the inferior colliculus and auditory cortex [4], [68], [69].

## Supporting Information

Text S1 **Model Appendix.** Equations governing intrinsic and synaptic characteristics of the MGB model neuron.
(DOC)
