# Supplementary material for: A Computational Model of Cellular Mechanisms of Temporal Coding in the Medial Geniculate Body (MGB)
Source: PLoS One. 2011 Dec 16;6(12):e29375. doi: 10.1371/journal.pone.0029375 (PMC3241713; doi:10.1371/journal.pone.0029375)
Supplement: Text S1 — Model Appendix. Equations governing intrinsic and synaptic characteristics of the MGB model neuron. (DOC) [file pone.0029375.s001.doc]

**Text S1: Model Appendix**

The ionic channels for the model are described by the following equation:

*I*x = gx(Vm – Ex)

where gx is the time-varying conductance for an individual ion channel multiplied by the difference between the membrane potential, Vm and the reversal potential for that ion channel, Ex. The time course and magnitude of the activation and inactivation parameters are given below. Equations for intrinsic currents are from previous modeling studies of Destexhe et al. [20], Huguenard and McCormick [19] and McCormick and Huguenard [18]. Equations for AMPA and NMDA currents are from experimental studies in the lateral geniculate and ventrobasal nucleus [26,40] and from the experimental data in Bartlett and Smith (2002) [11]. Equations for the magnitude and duration of short-term plasticity were estimated from the experimental data in Bartlett and Smith [11]. The models were implemented in NEURON v7.0 [36] using a time step of 0.02ms and a temperature of 34°C. Times are in ms, voltages are in mV, concentrations are in mM, and currents are in mA/cm2. Default model parameters are given in Table 2.

*Fast, transient Na+ current*

*I*Na = gNam3h(Vm – ENa)

v2 = v + 63

*τ*m = 1 / (αm + β)

m∞ = αm / (αm + βm)

αm = [0.32(– (Vm + 50))]/[e(-(Vm + 50)/4) – 1]

βm = [0.28 * ((Vm + 23) ]/[e((Vm + 23)/5) – 1]

αh = 0.128e(-(Vm + 46)/18)

βh = 4 /[1 + exp(-(Vm + 23)/5)]

*Delayed rectifier K+ current*

*I*Kdr = gKdrn4(Vm – EK)

αn = a = [0.032(– (Vm + 48))] / [ e(-(Vm + 48)/5) – 1]

βn = 0.5 * exp(-(Vm + 53)/40)

*Transient, depolarization-activated K+ current (IA)*

*I*A = gAm4h(Vm – EK)

m∞ = 1.0 / [1+e(-(Vm+60)/8.5)]

τm = [1.0/(e((Vm + 35.82)/19.69) +e(-(Vm + 79.69)/12.7)) + 0.37]

h∞ = 1.0 / [1+e((Vm + 78)/6)]

τh = 1.0/ [e((Vm + 46.05)/5) + e(-(Vm + 238.4)/37.45) ] for *Vm* < -63mV

τh = 19 for Vm ≥ -63mV

*Slowly inactivating, depolarization-activated K+ current (IK2)*

*I*k2 = gk2mh(Vm – EK)

m∞ = 1 / [1 + e( (-v-10)/17)]

τm = 4.95 + 0.5 / [e(( v - 81)/25.6) + e((- v - 132) / 18)]

h∞ = 1 / [1 + e( ( Vm + 58 ) / 10.6 ) ]

τh = 60 + 0.5 / [e( ( Vm - 1.33 ) / 200 ) + e( ( - Vm - 130 ) / 7.1 )]

*Persistent, depolarization-activated Na+ current (INap)*

*I*Nap = gNapm(Vm – ENa)

m∞ = 1/[1 + e((-49 - v)/5)]

αm = 0.091 * (v + 38) / (1 - exp(-(v + 38)/5))

βm = -0.062 * (v + 38) / (1 - exp((v +38)/5))

τm = 1/( αm + βm)

*Low-threshold Ca2+ current (IT)*

IT = PCaTm2hG(Vm,Cao,Cai)

G(V,Cao,Cai) = (Z2F2Vm/RT)[(Cai­-Caoe(-ZFVm/RT))/(1-e(-ZFVm/RT))]

m∞ = 1 / ( 1 + e(-(Vm+57)/6.2) )

h∞ = 1 / ( 1 + e((Vm+81)/4) )

τm = 0.612 + 1.0 / ( exp(-(v+132)/16.7) + exp((v+16.8)/18.2) )

τh = e((Vm+467)/66.6) for Vm < -80

τh = 28 + e(-(Vm+22)/10.5) for Vm ≥ -80

The expressions for activation and inactivations functions were obtained from Huguenard and McCormick [19], which were later modified by Destexhe et al. [20]. Calcium handling was modeled by a first-order representation of Ca2+ pumps and buffers [18] with a time constant of decay of Ca2+ of 5ms. The free intracellular Ca2+ concentration was 240nM and extracellular Ca2+ concentration was 2mM, which corresponds to reversal potential of approximately 120mV.

*High-threshold Ca2+ current (IL)*

IL = PCaLm2hG(Vm,Cao,Cai)

G(V,Cao,Cai) = (Z2F2Vm/RT)[(Cai­-Caoe(-ZFVm/RT))/(1-e(-ZFVm/RT))]

αm = 1.6/[1 + e(-0.072*(Vm - 5.0))]

βm = 0.02(Vm - 1.31)/[e((Vm - 1.31)/5.36) – 1]

m∞ = αm /(αm + βm)

τm = 1/(αm + βm)

*Hyperpolarization-activated cation current (Ih)*

*I*h = ghm(Vm - Eh)

m∞ = 1/[1+ e((Vm+75)/5.5)]

τm = 1/[e(-14.59 – 0.086Vm) + e(-1.87 + 0.0701Vm)]

*AMPA/NMDA receptor mediated synaptic current*

*I*AMPA = gAMPA (Vm – EAMPA)

gAMPA = gAMPAmax [1.43*(e(-t/6) - e(-t/.546))]

*I*NMDA = gNMDA (Vm – ENMDA)

gNMDA = gNMDAmax B*[2.5*(e(-t/40) -e(-t/13.2))]

B = 1/(1 + 0.28*e-0.062Vm)

*Short-term synaptic depression and facilitation*

$$W_{dep}=[1-\left(a_{d}e^{-t_{t-1}/\tau_{R}}-a_{d}e^{-t_{t-1}/\tau_{F}}\right)][1-\left(a_{d}e^{-t_{t-2}/\tau_{R}}-a_{d}e^{-t_{t-2}/\tau_{F}}\right)]$$

$$W_{fac}=[1+\left(a_{f}e^{-t_{t-1}/\tau_{R}}-a_{f}e^{-t_{t-1}/\tau_{F}}\right)]$$

Where a*d*, a*f*, R, and F are amplitude, rise and decay constants, respectively. The value of tt-1 and tt-2 refer to time from the previous EPSP and 2nd previous EPSP, respectively. The two previous spikes were included in determining synaptic depression because previous work showed that depression largely reached a steady state following two short intervals [11].

*Input Spike Probability*

Sprob = 1 – [(e-(ICI – 8)/2)/(1 + e-(ICI-8)/2)]

The above equation describes the input spike probability from central nucleus of inferior colliculus (ICc) that is based on data recorded from rats in response to sinusoidally amplitude-modulated tones [30] and cats in response to periodic noise bursts [31]. This produces reliable inputs at high ISIs with decreasing input probability with reduced ICI.
